# Protein disulfide isomerase ERp57 protects early muscle denervation in experimental ALS

**DOI:** 10.1186/s40478-020-01116-z

**Published:** 2021-02-04

**Authors:** Pablo Rozas, Cristina Pinto, Francisca Martínez Traub, Rodrigo Díaz, Viviana Pérez, Daniela Becerra, Patricia Ojeda, Jorge Ojeda, Madison T. Wright, Jessica Mella, Lars Plate, Juan Pablo Henríquez, Claudio Hetz, Danilo B. Medinas

**Affiliations:** 1 Biomedical Neuroscience Institute, Faculty of Medicine, University of Chile, Independencia 1027, P.O. Box 70086, Santiago, Chile; 2 Center for Geroscience, Brain Health and Metabolism, Santiago, Chile; 3 Program of Cellular and Molecular Biology, Institute of Biomedical Sciences, University of Chile, Santiago, Chile; 4 Neuromuscular Studies Laboratory (NeSt Lab), Department of Cell Biology, Faculty of Biological Sciences, Center for Advanced Microscopy (CMA Bio-Bio), Universidad de Concepción, Concepción, Chile; 5 Department of Chemistry and Department of Biological Sciences, Vanderbilt University, Nashville, TN USA; 6 Buck Institute for Research on Aging, Novato, CA USA

**Keywords:** Amyotrophic lateral sclerosis, Mutant SOD1, ERp57, Protein aggregation, Neuromuscular junction

## Abstract

Amyotrophic lateral sclerosis (ALS) is a progressive fatal neurodegenerative disease that affects motoneurons. Mutations in superoxide dismutase 1 (SOD1) have been described as a causative genetic factor for ALS. Mice overexpressing ALS-linked mutant SOD1 develop ALS symptoms accompanied by histopathological alterations and protein aggregation. The protein disulfide isomerase family member ERp57 is one of the main up-regulated proteins in tissue of ALS patients and mutant SOD1 mice, whereas point mutations in ERp57 were described as possible risk factors to develop the disease. ERp57 catalyzes disulfide bond formation and isomerization in the endoplasmic reticulum (ER), constituting a central component of protein quality control mechanisms. However, the actual contribution of ERp57 to ALS pathogenesis remained to be defined. Here, we studied the consequences of overexpressing ERp57 in experimental ALS using mutant SOD1 mice. Double transgenic SOD1^G93A^/ERp57^WT^ animals presented delayed deterioration of electrophysiological activity and maintained muscle innervation compared to single transgenic SOD1^G93A^ littermates at early-symptomatic stage, along with improved motor performance without affecting survival. The overexpression of ERp57 reduced mutant SOD1 aggregation, but only at disease end-stage, dissociating its role as an anti-aggregation factor from the protection of neuromuscular junctions. Instead, proteomic analysis revealed that the neuroprotective effects of ERp57 overexpression correlated with increased levels of synaptic and actin cytoskeleton proteins in the spinal cord. Taken together, our results suggest that ERp57 operates as a disease modifier at early stages by maintaining motoneuron connectivity.

## Introduction

Amyotrophic lateral sclerosis (ALS) is a progressive and fatal late-onset neurodegenerative disease characterized by loss of motoneurons leading to muscle weakness, paralysis and death [1]. Although most of ALS cases have no familial records being referred to as sporadic (sALS), around 10% are inherited and termed familial ALS (fALS) [2]. Genetic studies of fALS cases have led to the identification of causative mutations in several genes. The most common genetic alterations in ALS are the GGGGCC (G_4_C_2_) hexanucleotide repeat expansion in C*9ORF72*, nonsynonymous mutations in the genes encoding superoxide dismutase 1 (SOD1), trans-active response (TAR) DNA-binding protein 43 (TDP-43), and fused in sarcoma (FUS) [2, 3]. Mice overexpressing distinct ALS-linked mutant SOD1 develop progressive motor impairment with different degrees of severity depending on the specific mutation and transgene copy number [4]. Mutant SOD1^G93A^ transgenic mouse line is the most well-characterized preclinical ALS model because it recapitulates key disease features such as progressive decrease of motor performance, neuromuscular junction (NMJ) denervation, loss of spinal motoneurons concomitantly with astrogliosis and microgliosis, organelle dysfunction and presence of intracellular mutant SOD1 inclusions [5, 6]. In addition, misfolding and aggregation of wild-type SOD1 have been also reported in sALS cases [7, 8].

Independent studies identified two protein disulfide isomerase family members (PDIs), PDI and ERp57 (also known as PDIA3 or GRP58), among the main proteins induced in spinal cord of ALS rodents at different disease stages, suggesting that dysregulation of redox folding in the endoplasmic reticulum (ER) contributes to disease pathogenesis [9–11]. PDI and ERp57 were also found up-regulated in *post-mortem* spinal cord tissue of sALS patients [10, 12]. In addition, increased levels of PDI were also detected in cerebrospinal fluid of sALS patients [10], while a proteomic screening in blood cells revealed ERp57 as the most reliable biomarker of sALS progression [13].

We have previously reported missense mutations in *PDIA1* and *PDIA3* genes, which encode for PDI and ERp57, respectively, as risk factors to develop ALS [14]. Moreover, intronic SNPs in *PDIA1* gene have been associated to decreased survival of two different population of ALS patients [15, 16]. We discovered that these PDIs enhance neuritogenesis in motoneurons, a function impaired by the ALS-linked mutations [17]. Furthermore, ERp57 deficiency in the nervous system resulted in altered NMJs and impaired motor function in mice [17]. In addition, PDI nitrosylation and co-localization with protein inclusions in spinal motoneurons of sALS cases suggest compromised chaperone function possibly due to protein oxidation and aggregation [10, 18]. Overexpression of PDI and ERp57 has been proposed to provide protection in cell culture models of ALS by reducing aggregation of SOD1 and TDP-43, an activity lost by the ALS-linked PDI mutants [11, 19]. Finally, PDIs have been linked to the progression of other neurodegenerative diseases linked to protein misfolding including Alzheimer’s, Huntington’s, Parkinson’s and Prion-related diseases (reviewed in [20]), highlighting the protective effects of overexpressing ERp57 in cell culture and mice infected with prions [21, 22].

Despite the accumulating evidence linking PDIs to ALS pathogenesis, the actual contribution of these ER foldases to the disease process in vivo remains speculative. To date, no pharmacological or genetic studies have been reported to directly address the participation of PDIs in experimental ALS using preclinical models in mice. Here, we investigated the consequences of the artificial enforcement of ERp57 in the nervous system of mutant SOD1^G93A^ mice. Our data supports a protective role of ERp57 to motor function during early stages of ALS progression, preserving NMJ structure and delaying motor and electrophysiological impairment of affected muscles. Contrary to expectations, this motor unit protection did not correlate with a reduction in SOD1 aggregation. We speculate that experimental strategies to improve ER folding may translate into important beneficial effects to ALS patients.

## Materials and methods

### Animals

SOD1^G93A^ ALS mouse model carrying high copy number in C57BL/6 strain (B6.Cg-Tg (SOD1*^G93A^)1Gur/J) from Jackson Laboratory (strain number: 004435) was employed. SOD1^G93A^ transgene has human SOD1 promoter and approximately 25 copies inserted in tandem at mouse non-sexual chromosome 12 [4, 5, 23]. This promoter assures ubiquitous expression of human SOD1^G93A^. Importantly, this transgenic line is used in a heterozygous fashion recapitulating ALS features described previously [6]. Symptomatic mice were provided with pellet food on the floor of the cages in order to facilitate food intake. This was performed to reduce non-motor-related noise in disease progression parameters such as body weight and clinical score.

Mice from C57BL/6 strain that overexpress human form of wild-type ERp57 (termed ERp57^WT^) were generated in Centro de Estudios Científicos (CECs), Valdivia, Chile and characterized previously [24, 25]. This transgenic line employs the Prion protein promoter to express human ERp57 conjugated with a FLAG tag at the C-terminus.

All mice were housed in cages supplied with water and pellet food ad libitum in a light/dark cycle of 12 h/12 h at 22 ± 2 °C. General guidelines from the National Institutes of Health guide for the care and use of laboratory animals and from preclinical animal research in ALS/MND were followed [26]. The experimental procedures involving these mouse lines were approved by the Institutional Review Board for Animal Care of the Faculty of Medicine of the University of Chile (approved protocol CBA #0821-FMUCH). To generate double transgenic animals, heterozygous female mice from ERp57^WT^ colony were crossed with heterozygous male mice from SOD1^G93A^ line (see Additional file 1: Table S1).

### Phenotypic characterization

SOD1^G93A^ ALS model is characterized by progressive loss of body weight, due to muscle atrophy and impairment of muscles involved in feeding (chewing and swallowing, mainly), in addition to hindlimb muscle denervation and paralysis [6]. Disease progression was followed using wire hang test, body weight measurements, rotarod test, and clinical score analysis. Disease end point was considered as the time point when the mouse was unable to right itself within 10 s when put on its side.

Wire hang test consists on placing the mouse by its forepaws in a horizontal metal wire suspended by two vertical plastic bars 30 cm above the table surface (floor). Wire hang test score was determined as a function of mouse performance in trying to reach one of the vertical bars and then climbing down and reaching the floor within 30 s. The scale used to score performance was: 0: mouse falls on the floor before the first 10 s; 1: the mouse falls on the floor between 10 and 30 s; 2: the mouse tries to reach the horizontal bar using the hindlimbs without success; 3: the mouse reaches the horizontal bar with at least one of the hindlimbs; 4: the mouse reaches the horizontal bar with all limbs including the tail; 5: the mouse reaches and climbs on one of the vertical bars; 6: the mouse reaches the floor. Wire hang test was performed in a single session of 3 trials once a week starting at 6 weeks of age. Disease onset was defined as the time point when mice started to lose performance. Details for body weight measurements, rotarod test and clinical score analysis are provided in SI Materials and Methods.

### Electromyography (compound muscle action potential)

Male mice were anesthetized using isoflurane/oxygen mixture supplied by precision vaporizer RC2 Rodent Circuit Controller Anesthesia System (VetEquip Inc.). Gastrocnemius and tibialis muscles are well known to show denervation in mutant SOD1 mice models predicting onset of motor problems and survival [27]. Mice were laid on a non-conductive plastic procedure bed. Power lab 26T data acquisition system with LabChart software for data analysis (ADInstruments, New South Wales, Australia) was used. Two electrode pairs were used. One pair for recording and the other for delivering the electrical stimulus. For the recording electrode pair: a positive needle electrode was inserted intramuscularly to record compound muscle action potential (CMAP). Its ground electrode was placed subcutaneously in the ipsilateral paw. For stimulus electrode pair: ground electrode for the stimulus was inserted in the perianal region. The positive electrode for stimulation was manually placed on the surface of the skin (without insertion) at the lumbar spine region. The stimulation protocol consisted in a single stimulus of 20 mA given at the fifth second after protocol initiation. This latency time was used to ensure correct position of stimulation electrode. CMAP was calculated as the total amplitude of the sinusoidal recording (half period voltage amplitude). At least two sinusoidal responses were recorded at two different regions for each muscle. CMAP value for each muscle at each time point was defined as the maximum value obtained in that session, since it is interpreted as the maximum electrical response capacity of the motor units. Mice were closely observed after sessions to assure proper recovery. For CMAP time course experiment, the same hindlimb (right side) was assessed over time.

### Lumbrical muscle innervation analysis

The hindlimb lumbrical muscles were dissected as described [28]. Briefly, the hindlimb plant skin was removed and the flexor digitorum longus tendon was cut and removed together with the lumbrical muscles of each hindlimb. The tissue was pinned down in a Sylgard 184 silicone elastomer-covered petri dish and immersed in cold phosphate buffered saline (PBS), where the first to fourth deep lumbrical muscles were carefully dissected from the surrounding connective tissue under a dissection microscope. Muscles were fixed in 0.5% paraformaldehyde, permeabilized with PBS-T (0.5% Triton X-100 in PBS), blocked with 4% BSA in PBS-T (blocking solution) over night at 4 °C and then incubated with 1:300 anti-NF-M (Developmental Studies Hybridoma Bank, 2H3) and 1:50 anti-SV2 (Developmental Studies Hybridoma Bank, AB_2315387) antibodies diluted in blocking solution over night at 4 °C. After washing, samples were incubated with the corresponding 1:300 anti-donkey secondary antibody (Jackson) along with 1:500 Alexa-488 conjugated α-bungarotoxin during 2 h at room temperature (RT). The lumbrical muscles were post-fixed in 1% paraformaldehyde, washed and subsequently mounted in DAKO fluorescence medium. Confocal *z*-plane optical 1 μm sections were captured using inverted Zeiss LSM 780 multiphoton and LSM 700 laser scanning confocal microscopes (CMA BioBio facility, University of Concepcion, Chile). Confocal pre- and post-synaptic z-stack channels were projected, binarized, noise-reduced, and overlapped according to NMJ-morph guidelines [29]. Endplate area and overlap were quantified using “analyze particle” function from Fiji [30] setting a μm size threshold from 50 to infinity in order to automatize the process and make it unbiased. These same regions of interests were used to quantify the overlap with pre-synaptic signal. A number of 3 to 9 images per animal containing 10 to 36 endplates each were used. Gaussian distributions were fitted using GraphPad Prism 7.0 software.

### Lumbar spinal cord histological analysis

Mice were deeply anesthetized with ketamine/xylazine and perfused transcardially with 0.9% NaCl followed by 4% paraformaldehyde in PBS. Laminectomy was performed to dissect whole spinal cord. Using sciatic nerve as reference, L5 segment was sectioned with a blade followed by a second sectioning 5 mm rostral-ward the first section in order to obtain L5 to L2 region. Spinal cords were post-fixed in 4% paraformaldehyde in PBS for 24 h at 4 °C. Tissues were dehydrated in sucrose gradient (7.5–15–30% sucrose in PBS for 1 h each at RT). Dehydrated tissues were embedded in O.C.T. Compound (Sakura FineTek) and cryosectioned (Leica CM 1510S cryostat) at 25 μm per section.

For staining of misfolded SOD1 and FLAG positive cells, immunofluorescence (IF) using anti-FLAG antibody (Sigma, F7425) and C4F6 anti-SOD1 antibody (Medimabs, MM-0070-2-P), was performed. Spinal cord sections were mounted on Superfrost slides (VWR International). Epitope retrieval was performed using citrate buffer pH 6.0 for 20 min at 95 °C. Sections were blocked in 1% BSA diluted in 0.02% Triton X-100 in PBS (blocking buffer) for 1 h at RT and then incubated with 1:250 anti-FLAG and 1:100 C4F6 in blocking buffer overnight at 4 °C. Sections were then washed in PBS containing 0.2% Triton X-100 three times for 5 min each and incubated with 1:1000 anti-rabbit Alexa-488 and 1:1000 anti-mouse Alexa-568 conjugated secondary antibodies (Molecular Probes), and 1:5000 Hoechst 33342 (Molecular Probes) for nuclear staining, in blocking buffer for 2 h at RT. After four washes in PBS, sections were covered with coverslips using Fluoromount-G (Thermo Fisher Scientific) as mounting medium. Confocal microscopy (Nikon eclipse C2+) was used to obtain microphotographs.

For staining of vulnerable motoneurons, IF analysis using anti-ChAT antibody (Millipore, AB144P) and anti-MMP-9 antibody (Abcam, ab38898) was performed. Free-floating spinal cord sections were blocked in 5% donkey serum diluted in 0.05% Triton X-100 in PBS (donkey serum blocking buffer) for 1 h at RT and then incubated with 1:200 anti-ChAT and 1:200 anti-MMP-9 in donkey serum blocking buffer overnight at RT with gentle agitation. Sections were then washed in PBS four times for 10 min each and incubated with 1:1000 anti-goat Alexa 488 and 1:1000 anti-rabbit Alexa 564 conjugated secondary antibody (Molecular Probes), and 1:5000 Hoechst 33342 (Molecular Probes) for nuclear staining, in donkey serum blocking buffer for 3 h at RT. After four washes in PBS, sections were mounted on Superfrost slides (VWR International) and covered with coverslips using Fluoromount-G (Thermo Fisher Scientific) as mounting medium. Confocal microscopy (Nikon eclipse C2+) was used to obtain microphotography of both ventral horns per section. ChAT and MMP-9 positive motoneurons were manually counted.

### Cell culture and constructs

NSC-34 motoneuron-like cell line was obtained from Dr. Neil Cashman (University of British Columbia, Vancouver, Canada). Cells were cultured in DMEM supplemented with 1 mM pyruvate, 2 mM glutamine, 5% fetal bovine serum, and antibiotics (10,000 U/mL Penicillin, 10 µg/mL streptomycin), at 37 °C and 5% CO_2_. For SOD1^G93A^ aggregation assays, 250,000 cells per well were seeded in 6-well plates. Constructs for expression of C-terminus EGFP-tagged human SOD1 (SOD1-EGFP) and SOD1-EGFP targeted to secretory pathway (ER-SOD1-EGFP) containing superoxide dismutase 3 (SOD3, extracellular SOD) signal peptide in the protein N-terminus were generous gift from Dr. Julie Atkin (Macquarie University, Sydney, Australia) [31]. SOD1 constructs and C-terminus V5 tagged human ERp57 were transfected using Effectene reagent (Qiagen) following manufacturer’s instructions 24 h post seeding. The amount of plasmid used was 0.8 μg for each construct.

### Tissue homogenization and protein extracts

NSC-34 cells were harvested 48 h after transfection by resuspension and centrifugation (3000* g*, 5 min, 4 °C) following one wash in ice-cold PBS and cell pellets were kept frozen at − 80 °C until analysis. Animals were euthanized using CO_2_ chamber and lumbar spinal cord was dissected on ice and immediately stored at − 80 °C. Spinal cord tissue and NSC-34 cell pellet were homogenized in TEN buffer (10 mM Tris–HCl, 1 mM EDTA, 100 mM NaCl, pH 8.0) with proteases and phosphatases inhibitors (Roche). Homogenates were separated into two fractions: (1) for protein analysis, tissue and cells homogenates were diluted in TEN buffer with proteinase and phosphatase inhibitors plus 1% NP-40 and 50 mM iodoacetamide (to inhibit artificial disulfide bond formation); (2) for RNA analysis, tissue homogenates were diluted in TRIzol reagent (Thermo Fisher Scientific). Protein fractions were sonicated for 15 s and quantified using BCA protein assay (Thermo Fisher Scientific).

For SDS-PAGE and western blot analysis, protein samples were prepared using 100 mM DTT or deionized water (to assess the effect of disulfide bonds on protein aggregates) in 5× loading buffer (0.2 M Tris–HCl pH 6.8, 10% SDS, 0.05% bromophenol blue and 20% glycerol). Protein samples were incubated at 95 °C for 5 min before SDS-PAGE. Polyacrylamide gel electrophoresis was performed under denaturing conditions using molecular weight markers (Thermo Fisher Scientific) and proteins were electrotransferred onto PVDF membranes. The membranes were blocked in 5% non-fat dry milk in PBS (blocking solution) and primary antibodies were diluted in blocking solution and incubated over night at 4 °C. The following primary antibodies and dilutions were used: 1:3000 sheep anti-SOD1 (Merck, 574597); 1:1000 mouse anti-ERp57 (Abcam, ab13506); 1:1000 rabbit anti-ERp57 (Santa Cruz Biotechnology, SC-28823); 1:5000 mouse anti-V5 tag (Thermo Fisher Scientific, R960-25); 1:1000 rabbit anti-Marcks (Thermo Fisher Scientific, PA5-105296); 1:20,000 mouse anti-β actin (MP Biomedicals, C4). Membranes were washed thrice in 0.1% Tween in PBS (PBS-T) for 5 min each and incubated with the corresponding 1:2000 HRP-conjugated secondary antibodies (Life Technologies) in blocking buffer for 2 h at RT. After washing thrice in PBS-T, the western blot was developed using the ECL method (Thermo Fisher Scientific) following manufacturer’s instructions. Chemiluminescence signal and protein ladder images were acquired using ChemiDoc Imaging System (BioRad).

For filter trap, sonicated protein fractions were treated with 100 mM DTT or deionized water for 30 min on ice and then diluted in PBS containing 1% SDS (PBS-SDS). Final protein concentration in PBS-SDS was 0.25 μg/μL to avoid artificial clumping of the membrane pores. Protein samples were vacuum filtered through a cellulose acetate membrane with 0.22 μm pore size. Membrane was then washed once with PBS-SDS and twice with PBS-T for 5 min at RT each and blocked with 5% non-fat dry milk in PBS for 30 min at RT. Membrane was incubated with 1:3000 sheep anti-SOD1 (Merck, 574597) primary antibody over night at 4 °C diluted in blocking buffer. Membranes were washed thrice in PBS-T for 5 min each and incubated with 1:2000 anti-sheep HRP-conjugated secondary antibody (Life Technologies) in blocking buffer for 2 h at RT. After washing in PBS-T, filter trap was developed using ECL method (Thermo Fisher Scientific) following manufacturer’s instructions. Chemiluminescence signal was detected using ChemiDoc Imaging System (BioRad).

For analysis of disulfide-dependent high molecular weight (HMW) protein aggregates, protein extracts alkylated with iodoacetamide were treated or not with 100 mM of the thiol reducing agent dithiothreitol (DTT) in TEN buffer supplemented with 50 mM Tris–HCl pH 8.0 for 30 min on ice. Samples were diluted in Laemmli’s loading buffer in the absence or presence of 100 mM of DTT and incubated 5 min at 95 °C. Then, the samples under non-reducing and reducing conditions were run separately on SDS-PAGE mini gels at 80 V. Before electroblotting of proteins on PVDF membranes, the gels were incubated for 30 min in SDS-PAGE running buffer containing 50 mM DTT under gentle agitation to assure even transfer of disulfide reduced and oxidized proteins. Membranes were then submitted to western blot procedures described above.

### Quantitative real-time PCR analysis

For RT-qPCR, a total of 1 μg RNA was isolated from tissue using TRIzol reagent (Thermo Fisher Scientific) following manufacturer’s instructions. cDNA was synthesized with SuperScript III (Thermo Fisher Scientific) using random primers p(dN)6 (Roche) according to manufacturer’s instructions. Quantitative real-time PCR (qPCR) reactions employed EvaGreen™ reagent (Biotium) in a mix of 4 μL of 1:20 cDNA: nuclease-free water dilution, 0.5 μL of 10 μM primers, 10 μL of EvaGreen™ and 7.5 μL of nuclease-free water in a final volume of 20 μL. qPCR was performed in Stratagene Mx3000P system (Agilent Technologies). Thermal profile used for qPCR was: 1 denaturing cycle of 95 °C for 10 s; 40 amplification cycles of 95 °C for 15 s, 60 °C for 18 s, 72 °C for 15 s; 1 final amplification cycle of 95 °C for 15 s, 25 °C for 1 s, 70 °C for 15 s and 95 °C for 1 s. The relative amounts of mRNAs were calculated from the values of comparative threshold cycle by using *Actin* mRNA as control. Primer sequences: *SOD1*: forward 5′-CATCAGCCCTAATCCATCTGA-3′ and reverse 5′-CGCGACTAACAATCAAAGTGA-3′; *ERp57*: forward 5′-GTCATAGCCAAGATGGATGCC-3′ and reverse 5′-TTAATTCACGGCCACCTTCATA-3′; *Xbp1s*: forward 5′-TGCTGAGTCCGCAGCAGGTG-3′ and reverse 5′-GACTAGCAGACTCTGGGGAAG-3′; *Chop*: forward 5′-GTCCCTAGCTTGGCTGACAGA-3′ and reverse 5′-TGGAGAGCGAGGGCTTTG-3′; *Edem1*: forward 5′-AAGCCCTCTGGAACTTGCG-3′ and reverse 5′-AACCCAATGGCCTGTCTGG-3′; Pdia1: forward 5′-CAAGATCAAGCCCCACCTGAT-3′ and reverse 5′-AGTTCGCCCCAACCAGTACTT-3′; *Actin*: forward 5′-CTCAGGAGGAGCAATGATCTTGAT-3′ and reverse 5′-TACCACCATGTACCCAGGCA-3′.

### Quantitative proteomic analysis

Lumbar spinal cord tissue was homogenized in TEN buffer as described above. For each sample, 10 μg of lysate was precipitated with chloroform/methanol. Samples for mass spectrometry analysis were prepared as described [32]. Air-dried pellets were resuspended in 1% RapiGest SF (Waters) and diluted to final volume in 100 mM HEPES (pH 8.0). Proteins were reduced with 5 mM Tris(2-carboxyethyl)phosphine hydrochloride (Thermo Fisher Scientific) for 30 min and alkylated with 10 mM iodoacetamide (Sigma Aldrich) for 30 min at room temperature in the dark. Proteins were digested for 18 h at 37 °C with 0.5 μg trypsin (Thermo Fisher Scientific). After digestion, the peptides from each sample were reacted for 1 h with the appropriate tandem mass tag (TMTpro 16plex) isobaric reagent (Thermo Fisher Scientific) in 40% (v/v) anhydrous acetonitrile and quenched with 0.4% ammonium bicarbonate for 1 h. Samples with different TMT labels were pooled and acidified with 5% formic acid. Acetonitrile was evaporated on a SpeedVac and debris removed by centrifugation for 30 min at 18,000* g*. MudPIT microcolumns were prepared as described [33]. LC–MS/MS analysis was performed using a Exploris 480 mass spectrometer equipped with an Ultimate 3000 nLC 1000 (Thermo Fisher Scientific). MudPIT experiments were performed by 10 μL sequential injections of 0, 10, 20, 30, …, 100% buffer C (500 mM ammonium acetate in buffer A) and a final step of 90% buffer C/10% buffer B (100% acetonitrile, 0.1% formic acid, v/v/v) and each step followed by a gradient from buffer A (95% water, 5% acetonitrile, 0.1% formic acid) to buffer B. Electrospray was performed directly from the analytical column by applying a voltage of 2.2 kV with an inlet capillary temperature of 275 °C. Data-dependent acquisition of MS/MS spectra was performed with the following settings: eluted peptides were scanned from 375 to 100 m/z with a resolution of 120,000. Precursor ions from full scans were fragmented under TopSpeed setting using a cycle time of 3 s, HCD collision energy of 29%, isolation window of 0.4 m/z, a resolution of 30,000 using TurboTMT option, normalized ACG target 200%, maximum IT 120 ms, and scanned with first mass at 110 m/z. Dynamic exclusion was set to 10 s. Peptide identification and protein quantification was performed using Proteome Discoverer 2.4 (Thermo Fisher Scientific). Spectra were searched using SEQUEST against a UniProt mouse proteome database (accessed on Nov. 2019). Searches were carried out using a decoy database of reversed peptide sequences using Percolator node for filtering and the following settings: 20 ppm peptide precursor tolerance, 6 amino acid minimum peptide length, trypsin cleavage (2 missed cleavage events), static Cys modification of 57.0215 (carbamidomethylation), variable Met oxidation, and static N-terminal and Lys modification of 304.207 (TMTpro 16plex), FDR 0.01, and a minimum of 2 peptide IDs per protein. Normalization of TMT reporter ion intensities was carried out based on total peptide abundance in each channel, and subsequently, TMT intensity ratios for each identified protein were calculated between sample groups: Non-Tg (n = 4), ERp57^WT^ (n = 4), SOD1^G93A^ (n = 4) and SOD1^G93A^/ERp57^WT^ (n = 3). TMT intensities were log2-transformed to calculate abundance differences. Significance was assessed by multiple two-tailed unpaired t-tests using the FDR approach and two-stage step-up method of Benjamini, Krieger, and Yekutieli with Q = 5% in Graphpad Prism 8.4. Raw data along with ERp57 peptides analysis are provided in Additional file 2: Table S2.

### Cell culture neuritogenesis assay

NSC-34 cell line expressing wild-type SOD1 (SOD1^WT^) or mutant SOD1 (SOD1^G93A^) in a stable form were maintained in proliferation medium composed of DMEM, 4.5 g/L Glucose, 8 mM l-Glutamine (HyClone), antibiotics (10,000 U/mL Penicillin, 10 µg/mL streptomycin) (Biological Industries), 15% fetal bovine serum and 0.4 mg/mL G418 (Merck) as selection antibiotic for the plasmid [34].

For neuritogenesis assay, NSC-34 cells were grown on 18 × 18 mm glass coverslips and incubated in OptiMEM medium (Invitrogen) and transfected using a Lipofectamine Plus Reagent mix (Invitrogen), according to manufacturer’s instructions for 24 h. The amounts of plasmid used were: 0.8 μg of YFP as control or 0.8 μg of human ERp57^WT^-V5 tagged. Both plasmids were in pcDNA3.1 backbone. Cells were rinsed once with PBS and induced to differentiate using Neurobasal medium (Invitrogen) without FBS for 24 h. To identify cells expressing ERp57, anti-V5 (Thermo Fisher Scientific, R960-25) immunostaining was performed. The medium was removed and cells were rinsed with cold PBS, fixed with 4% paraformaldehyde in PBS for 30 min at 4 °C and subsequently permeabilized with 0.1% Triton X-100 in Tris-buffered saline (TBS). Cells were washed with TBS and then incubated with 1:1000 anti-V5 primary antibody diluted in 1% BSA in TBS, for 15 h at 4 °C. Coverslips were incubated for 2 h at room temperature with anti-mouse Alexa-488 conjugated secondary antibody (Invitrogen) in 1% BSA in TBS. After 3 washes, nuclei were labeled with DAPI and coverslip mounted with Faramount mounting medium (Dako). Images were acquired with a laser confocal LSM780 Zeiss microscope. Acquired images were analyzed using ImageJ software. The number of differentiated cells was determined considering cells having at least one neurite with a minimum size equal to the cell soma diameter. For each condition, 10 fields from 3 different experiments were quantified.

### Statistical analysis

Statistics were performed using Graphpad Prism 7.0 (GraphPad Software). Data were compared using One-way ANOVA or Two-way ANOVA for unpaired groups followed by multiple comparison post-test to compare more than two groups as stated in each figure. Student’s *t*-test was performed for unpaired group comparison between two groups; Log-rank test was performed to evaluate significance in Kaplan–Meier survival curves. Statistical analysis of Gaussian fits for NMJ overlap analysis was performed using non-linear fit followed by extra sum-of-squares F test. For proteomic experiment, statistical analysis was performed using multiple t-test with two-stage step-up method of Benjamini, Krieger and Yekutieli, with a False Discovery Rate of 5% in Graphpad prism 8.4. In all plots, *p* values are shown as indicated: *p ≤ 0.05, **p ≤ 0.01 and ***p ≤ 0.001 and were considered significant.

## Results

### ERp57 overexpression improves motor function of mutant SOD1^G93A^ mice at early-symptomatic stage

**Fig. 1:**
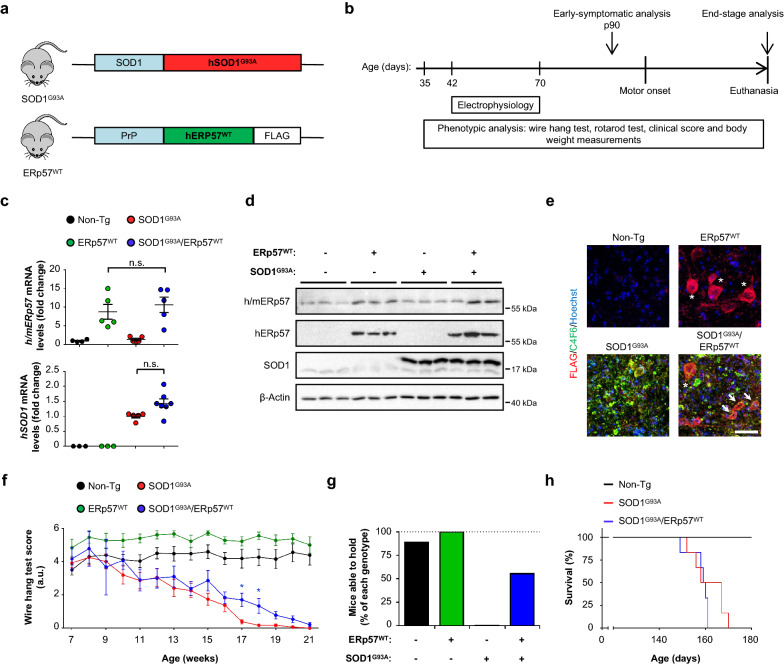
ERp57 improves motor performance of mutant SOD1 mice. **a** SOD1^G93A^/ERp57^WT^ double transgenic mice were generated by crossing heterozygous mice of SOD1^G93A^ and ERp57^WT^ transgenic lines. The mutant SOD1 mouse line overexpresses human SOD1^G93A^ driven by SOD1 promoter. The ERp57^WT^ mouse line overexpresses wild-type human ERp57 conjugated with a FLAG tag at the C-terminus driven by Prion protein promoter. **b** Schematic view of the protocol for characterization of SOD1^G93A^/ERp57^WT^ mice, which were monitored weekly through their lifespan to assess several phenotypic parameters, including body weight measurements, motor performance in wire hang test and rotarod test, and clinical score. Electrophysiological activity was evaluated as compound muscle action potential (CMAP). **c** mRNA levels of human *SOD1* (*hSOD1*) and human and endogenous mouse *ERp57 (h/mERp57*) were measured by RT-qPCR in lumbar spinal cord. Statistical analysis was performed using one-way ANOVA with Tukey’s multiple comparison test. Mean ± S.E. is shown; n.s., *p* > 0.05 (n = 3–7 mice per genotype). Each data point represents one animal. **d** Protein levels of ERp57 and SOD1 in lumbar spinal cord. h/m: human and mouse ERp57 forms. h: human ERp57 form. SOD1 western blot shows human (transgene; upper band) and mouse (endogenous; lower band) SOD1 forms. Each lane corresponds to one animal. **e** Representative immunofluorescence micrographs of ventral horns of the lumbar spinal cord stained against FLAG epitope in ERp57^WT^ construct (pseudocolored red) and misfolded human SOD1 (clone C4F6, pseudocolored green). One ventral horn is shown. Asterisk (*) indicates motoneurons discriminated by morphological and anatomical location in the ventral horn. Arrows indicate FLAG-positive cells containing misfolded SOD1. Nuclei stained with Hoechst 33,342 (pseudocolored blue). Scale bar: 50 μm. **f** Mice motor performance was assessed by wire hang test. Statistical analysis was performed using two-way ANOVA with Tukey’s multiple comparison test. Mean ± S.E. is shown; *p* values for comparison between SOD1^G93A^ and SOD1^G93A^/ERp57^WT^ are shown: *, *p* ≤ 0.05 (n = 6–14 male mice per genotype). **g** Percentage of mice shown in **e** able to hold themselves on the wire hang test for more than 10 s at symptomatic stage (17 weeks of age). **h** Survival of double transgenic mice compared to SOD1^G93A^ transgenic group. Non-transgenic (Non-Tg) littermates were plotted as control (n = 6 male mice per genotype)

To define the functional impact of increasing ERp57 levels on ALS onset and progression, we crossed SOD1^G93A^ mice with a transgenic line overexpressing ERp57^WT^ under the Prion promoter previously generated in our laboratory [24, 25] to obtain SOD1^G93A^/ERp57^WT^ double transgenic mice and control littermates (Fig. 1a). These animals were viable and born at Mendelian ratio (Additional file 1: Table S1). Mice were monitored until end-stage to assess disease signs and determine lifespan, in addition to perform histopathological and biochemical analysis (Fig. 1b). We confirmed equivalent expression levels of SOD1 and ERp57 transgenes in double transgenic animals when compared to single transgenic littermates at both mRNA and protein levels (Fig. 1c, d). Histological analysis of spinal cord ventral horn indicated the expression of FLAG-tagged ERp57 in motoneurons accumulating misfolded SOD1 (Fig. 1e), consistent with the previously reported neuronal expression of the transgene [24, 25].

Wire hang test was employed to monitor motor capacity over the course of the disease. SOD1^G93A^ mice showed a progressive decrease of performance, associated with loss of coordination, equilibrium, and strength (Fig. 1f and Additional file 1: Fig. S1a). Double transgenic male mice had significant better performance in this test during the symptomatic period (Fig. 1f), indicating protective effects of enforcing ERp57 expression in ALS. At 17 and 18 weeks of age, over half of the double transgenic mice were able to hold themselves on the wire while their SOD1^G93A^ counterparts fell on the floor before 10 s (Fig. 1g). Interestingly, ERp57^WT^ single transgenic mice performed better than non-Tg littermates for several weeks, supporting the notion that ERp57 can boost motor performance, even in non-diseased animals (Fig. 1f). However, double transgenic mice had no difference in other global disease parameters compared to SOD1^G93A^ mice (Additional file 1: Fig. S1b–f). Despite predictions that ERp57 overexpression would extend lifespan in ALS [19], SOD1^G93A^ and double transgenic mice showed the same survival rate (Fig. 1h and Additional file 1: Fig. S1c), suggesting that ERp57 operates as an ALS modifier at early-symptomatic stages impacting motor performance.

### ERp57 overexpression reduces mutant SOD1 aggregation at late disease stage

**Fig. 2:**
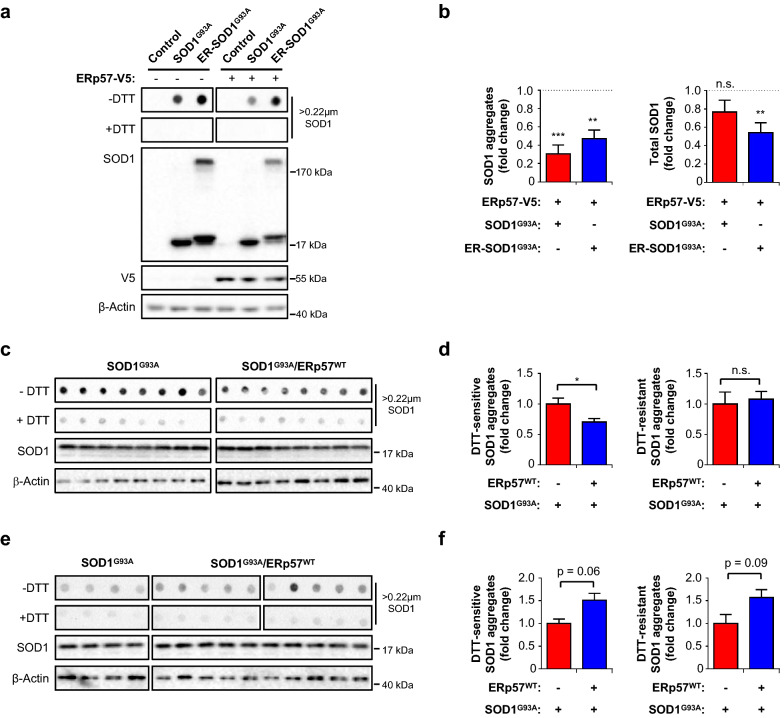
ERp57 overexpression reduces mutant SOD1 aggregation at the disease end-stage. **a** NSC-34 cells were co-transfected with constructs to express SOD1^G93A^-EGFP or SOD1^G93A^-EGFP tagged to the ER (ER-SOD1^G93A^) and ERp57-V5 tag or empty vector (mock). Filter-trap analysis under non-reducing (−DTT) and reducing (+DTT) conditions was performed 48 h after transfection for detection of SOD1 aggregates. Total SOD1 levels were assessed using western blot analysis under reducing (+DTT) conditions. Control: cells transfected with empty vector for SOD1 constructs. **b** Quantification of **a**. Results are plotted as fold change relative to mock transfected cells (dotted line). β-actin was employed as loading control. Statistical analysis was performed using Student’s *t*-test to compare against mock transfected cells. Mean ± S.E. is shown; *p* values: n.s., *p* > 0.05; **, *p* ≤ 0.01; and ***, *p* ≤ 0.001 (n = 6 independent experiments). **c** Filter-trap analysis of end-stage lumbar spinal cord extracts under non-reducing (−DTT) and reducing (+DTT) conditions for detection of SOD1 aggregates. β-actin and SOD1 western blot were employed as loading controls. **d** Quantification of **c**. DTT resistant aggregates were calculated directly from +DTT signal. DTT-sensitive aggregates were calculated as –DTT minus +DTT signal. Statistical analysis was performed using Student’s *t*-test. Mean ± S.E. is shown; *p* values: n.s., *p* > 0.05 and *, *p* ≤ 0.05 (n = 8–11 animals per genotype). **e** Filter-trap analysis of post-natal day 90 lumbar spinal cord extracts under non-reducing (−DTT) and reducing (+DTT) conditions to detect SOD1 aggregates at early-symptomatic stage. β-actin and SOD1 western blots were employed as loading controls. **f** Quantification of **e**. DTT resistant aggregates were calculated directly from +DTT signal. DTT-sensitive aggregates were calculated as –DTT minus +DTT signal. Statistical analysis was performed using Student’s *t*-test. Mean ± S.E. is shown (n = 4–10 animals per genotype). For **c** and **e**, each lane and dot corresponds to one animal

Mutant SOD1 is prone to misfold and form oligomers (high molecular weight (HMW) species) and large aggregates (larger than 0.22 μm in diameter). ERp57 has been proposed as a molecular chaperone able to reduce toxic aggregated forms of SOD1 due to its disulfide isomerase activity [11, 19]. ERp57 is mainly located in the ER, with a pool of the protein also residing in the plasma membrane [35]. On the other hand, SOD1 is a cytosolic protein that can be distributed to different subcellular locations including the ER [36–38]. However, the possible effects of ERp57 over SOD1 aggregates formation or clearance remain undefined in vivo. Thus, we determined if ERp57 overexpression influences SOD1^G93A^ aggregation using cell culture and our mouse model through the biochemical analysis of protein extracts under reducing and non-reducing conditions (with addition of the thiol reductant dithiothreitol, DTT) to discriminate disulfide-crosslinked species. We first performed experiments using the motoneuron-like NSC-34 cell line [39]. We transfected cells with constructs to express SOD1^G93A^ or ER-SOD1^G93A^, an ER-targeted version of the protein, to examine the contribution of the subcellular localization on protein aggregation. In line with previous observations [19], ERp57 overexpression reduced DTT-sensitive SOD1^G93A^ large aggregates as detected by filter-trap analysis (Fig. 2a, b). Moreover, the localization of SOD1^G93A^ to the ER favored protein aggregation through disulfide-crosslinks (Fig. 2a, b). ERp57 overexpression markedly decreased both aggregated and total levels of ER-SOD1^G93A^, supporting its function on quality control of misfolded and aggregated SOD1^G93A^ in the ER (Fig. 2b).

The analysis of SOD1^G93A^ aggregates in the spinal cord of double transgenic mice at different disease stages revealed a complex scenario in vivo. Mutant SOD1 aggregation and accumulation in the ER increases over the course of the disease [36, 40–43]. Thus, we measured SOD1^G93A^ aggregate levels in mice at end-stage. Consistent with our results in cell culture, filter-trap and western blot analysis showed that ERp57 overexpression reduces DTT-sensitive mutant SOD1 aggregates in terminally ill animals (Fig. 2c, d and Additional file 1: Fig. S2). To verify whether improved motor function of double transgenic mice was correlated with a reduction of SOD1 aggregates, we examined samples of mice at early-symptomatic stage (Fig. 2e). Unexpectedly, we observed a trend to augmented levels of aggregated SOD1 in double transgenic mice at this temporal window (Fig. 2f). Additionally, UPR activation was not observed at this disease stage as assessed by real time PCR of classical makers *Xbp1*s and *Chop* (Additional file 1: Fig. S3). Taken together, our results suggest that the effects of ERp57 overexpression on motor function are not due to a reduction of mutant SOD1 aggregation, and that ERp57 may act on aggregates accumulating at ER lumen only at advanced disease stage.

### Late motoneuron loss and neuroinflammation are unaffected in spinal cord of double transgenic mice

We assessed motoneuron number using anti-choline acetyltransferase (ChAT) staining in the lumbar segment of the spinal cord spanning L5-L2 regions, which corresponds to the primary affected zone in the SOD1^G93A^ mouse model [44]. ERp57^WT^ transgenic mice had the same number of motoneurons as non-transgenic littermates (Additional file 1: Fig. S4a). SOD1^G93A^ and double transgenic mice presented around 50% motoneuron loss at end-stage compared to non-diseased controls.

Microgliosis and astrogliosis are common histopathological features of ALS induced at symptomatic stages [2, 3]. These two parameters were assessed in end-stage mice using anti-Iba1 and anti-GFAP staining, respectively (Additional file 1: Fig. S4b–c). There was an increase of microgliosis and astrogliosis to the same extent in lumbar spinal cord of SOD1^G93A^ and double transgenic mice measured as percentage of ventral horn area stained with the glial marker. In addition, basal levels of Iba1 or GFAP staining were not modulated by ERp57 overexpression. These results suggest that ERp57 might affect the functionality of motoneurons rather than improving their viability or the proinflammatory environment in the spinal cord tissue.

### ERp57 overexpression delays electrophysiological impairment in SOD1^G93A^ mice hindlimbs

**Fig. 3:**
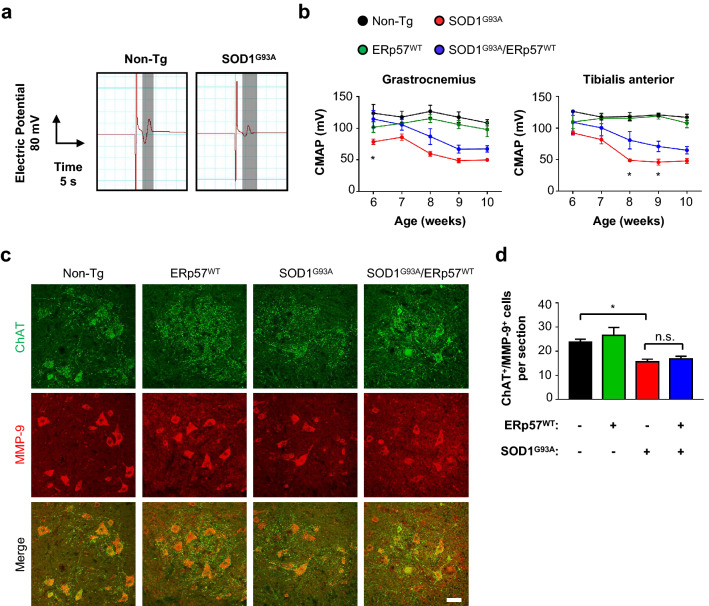
ERp57 overexpression improves hindlimb electrophysiology in mutant SOD1 mice. **a** Example of electrophysiological recordings of compound muscle action potential (CMAP) in gastrocnemius muscle of non-Tg and SOD1^G93A^ mice at post-natal day 90. Grey area shows CMAP signal after stimulation (first peak). **b** CMAP amplitude during early-symptomatic window. Statistical analysis was performed using two-way ANOVA with Tukey’s multiple comparison test. Mean ± S.E. is shown; *p* values for comparison between SOD1G93A and SOD1G93A/ERp57WT are shown: (“G93A” and “WT” in super index) *, *p* ≤ 0.05 (n = 3–7 animals per genotype). **c** Representative immunofluorescence micrographs of lumbar spinal cord sections stained with anti-ChAT and anti-MMP9 antibody at post-natal day 70. Ventral horns are shown. Scale bar: 50 μm. **d** Analysis of ChAT^+^/MMP9^+^ vulnerable motoneurons number in lumbar spinal cord quantified from at least 4 serial sections per animal. Statistical analysis was performed using one-way ANOVA with Tukey’s multiple comparison test. Mean ± S.E. is shown; *p* values: n.s., *p* > 0.05; *, *p* ≤ 0.05 (n = 3–7 animals per genotype)

Loss of NMJ integrity due to motoneuron denervation is an early pathogenic event in ALS patients and mouse models, representing a key parameter for ALS diagnosis [45]. Denervation occurs before the symptomatic stage and translates into reduced electric potential in affected muscles [27]. We measured compound muscle action potential (CMAP) in gastrocnemius and tibialis anterior before onset of motor problems (from 44 to 72 days old). CMAP is the addition of action potentials at the muscle in response to non-invasive spinal cord electrical stimulation, and decreasing values reflect NMJ denervation in the mutant SOD1^G93A^ mouse model [27] (Fig. 3a). SOD1^G93A^ mice displayed a decline in CMAP values in both muscles before appearance of motor problems as detected by wire hang test (Fig. 3b). Double transgenic mice presented consistently higher CMAP values than SOD1^G93A^ single transgenic littermates in gastrocnemius and tibialis anterior muscles at this disease time point, suggesting a protective role for ERp57 in maintaining skeletal muscle innervation (Fig. 3b).

To address if CMAP differences were the result of the loss of vulnerable motoneurons in the spinal cord, we performed immunostaining using anti-ChAT along with anti-matrix metalloproteinase 9 (MMP9) [46] (Fig. 3c). In line with our results at end-stage, ERp57 overexpression did not affect MMP9^+^ vulnerable motoneuron count in early-symptomatic mice, suggesting that ERp57 influences motoneuron function and possibly NMJ integrity rather than neuronal survival.

### ERp57 overexpression preserves hindlimb muscle innervation in SOD1^G93A^ mice

**Fig. 4:**
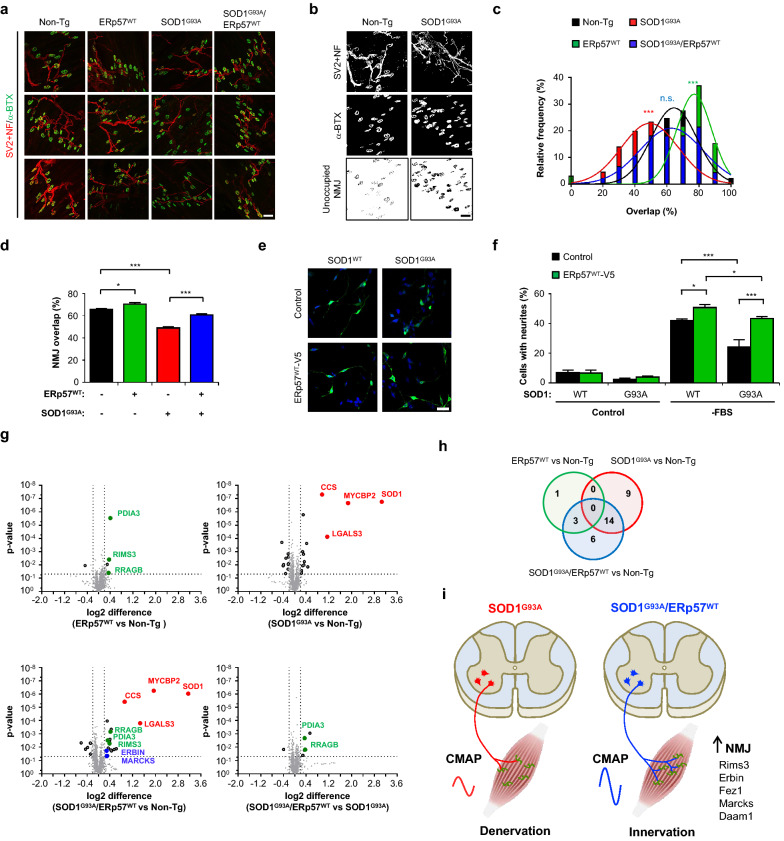
ERp57 preserves neuromuscular junction connectivity of ALS mice. **a** Neuromuscular junction (NMJ) staining of lumbrical muscle at post-natal day 90. Anti-SV2 and anti-NF-M staining correspond to pre-synaptic component (pseudocolored red). α-Bungarotoxin coupled to Alexa 488 (pseudocolored green) corresponds to post-synaptic endplate. Representative confocal optical sections of three animals per genotype are shown. Scale bar: 50 μm. **b** Analysis of NMJ integrity of lumbrical muscle at post-natal day 90. Confocal optical sections of pre- and post-synaptic components were binarized and automatically subtracted to quantify endplate area without pre-synaptic component (unoccupied NMJ). Representative confocal optical sections of non-Tg and SOD1^G93A^ mice are shown. Scale bar: 50 μm. **c** Distribution of NMJ occupied area (as percentage of endplate area) in lumbrical muscle at post-natal day 90. NMJ overlap histograms with Gaussian fits are shown. Statistical analysis was performed using non-linear fit followed by extra sum-of-squares F test comparing different genotype curve fits to non-Tg fit. *p* values: n.s., *p* > 0.05; ***, *p* ≤ 0.001 (n = 4 animals per genotype with 65–201 NMJ per animal quantified). **d** Analysis of **c** showing NMJ occupied area as average percentage of total endplate areas. Statistical analysis was performed using one-way ANOVA with Tukey’s multiple comparison test. Mean ± S.E. is shown; *p* values: *, *p* ≤ 0.05; ***, *p* ≤ 0.001 (n = 4 animals per genotype with 65–201 NMJ per animal quantified). **e** NSC-34 cell lines stably expressing wild-type SOD1 (SOD1^WT^) or mutant SOD1 (SOD1^G93A^) were transfected with constructs to express wild-type human ERp57 coupled to V5 tag (ERp57^WT^-V5) or YFP (Control, pseudocolored green). Cells were differentiated by serum deprivation for 24 h. Immunofluorescence staining against V5 tag was performed (pseudocolored green) along with Hoechst 33,342 staining (pseudocolored blue). Scale bar: 20 μm. **f** Analysis of neurite sprout of cells described in **e**. –FBS: serum deprivation. Statistical analysis was performed using one-way ANOVA with Tukey’s multiple comparison test. Mean ± S.E. is shown; *p* values: n.s., *p* > 0.05; *, *p* ≤ 0.05; ***, *p* ≤ 0.001 (n = 3 independent experiments). **g** Volcano plots of proteomic analysis of lumbar spinal cord at post-natal day 90. Each panel shows a different comparison between genotypes. Statistical analysis was performed using multiple t-test with two-stage step-up method using Benjamini, Krieger and Yekutieli approach with a False Discovery Rate of 5%. Hits with q-value ≤ 0.05 and *p* value ≤ 0.05 are highlighted on each plot (grey dots and black border). Selected hits with q-value ≤ 0.05 and *p* value ≤ 0.05 that are contributions from each genotype are highlighted on each plot (ERp57^WT^: solid green, SOD1^G93A^: solid red, SOD1^G93A^/ERp57^WT^: solid blue) (n = 3–4 animals per genotype). **h** Venn diagram of proteomic hits from genotype pair comparisons. Hits with q-value ≤ 0.05 and *p* value ≤ 0.05 were considered for analysis. **i** Schematic representation of ERp57 involvement in molecular and cellular pathways of ALS pathophysiology

To investigate if the protection of motor units in hindlimb muscles by ERp57 overexpression was due to NMJ maintenance, we performed morphological analysis of lumbrical muscle at early-symptomatic stage. This distal muscle represents an ideal source for motor unit information in the mutant SOD1 mouse model since it suffers denervation at pre-symptomatic stages and has a thin and flat anatomy that enables *en face* quantification of almost the entire content of NMJs [47]. We assessed NMJ innervation by measuring the overlap between pre-synaptic anti-neurofilament (NF)/anti-synaptic vesicle protein 2 (SV2) and post-synaptic α-bungarotoxin staining (Fig. 4a, b) [29]. Early-symptomatic SOD1^G93A^ transgenic mice had reduced innervation levels compared to non-Tg littermates (Fig. 4c, d), a phenomenon prevented in double transgenic mice as shown by significantly higher occupancy of lumbrical muscle endplates (Fig. 4c, d). Furthermore, ERp57 overexpression also increased the overlap between pre- and post-synaptic markers in non-diseased animals (Fig. 4c, d), suggesting a physiological role at basal levels. Overall, this data indicates that ERp57 overexpression enhances NMJ innervation, contributing to its maintenance at early-symptomatic stages of experimental ALS.

To further explore the significance of ERp57 as a protective factor supporting motoneuron connectivity in ALS, we studied neurite outgrowth in NSC-34 cells stably expressing wild-type SOD1 (SOD1^WT^) or SOD1^G93A^. We transiently transfected SOD1^WT^ or SOD1^G93A^ NSC-34 cells with constructs to express human ERp57 coupled to V5 tag (ERp57^WT^-V5) or YFP as control. Neuritogenesis was induced by serum deprivation for 24 h and the percentage of cells with neurites was quantified (Fig. 4e, f). As we previously reported [34], mutant SOD1^G93A^ decreased the number of cells with neurites compared to SOD1^WT^. ERp57 fully rescued neuritogenesis in NSC-34 cells overexpressing SOD1^G93A^. In addition, ERp57 overexpression increased basal neuritogenesis in SOD1^WT^ NSC-34 control cells, consistent with our previous findings [17].

To identify possible molecular mechanisms associated to the neuroprotection exerted by ERp57 overexpression in the mutant SOD1 mice, we performed quantitative proteomics of lumbar spinal cord tissue derived from early-symptomatic animals. This analysis corroborated similar overexpression levels of ERp57 and SOD1 in double transgenic mice compared to single transgenic littermates, along with induction of endogenous copper chaperone for SOD1 (Ccs) in ALS mice (Fig. 4g, Additional file 1: Fig. S5 and Additional file 2: Table S2). At this disease stage, the most prominent proteomic alterations detected in the mutant SOD1 model reflected protective pathways, with up-regulation of the atypical E3 ubiquitin-protein ligase Myc-binding protein 2 (Mycbp2) and Galectin-3 (Lgals3) (Fig. 4g and Additional file 1: Fig. S6). Mycbp2 participates in axonal growth and synaptogenesis [48, 49] and is up-regulated in the brain cortex of sALS patients [50]. Galectin-3 has been previously identified as a major proteomic hit up-regulated in tissue of transgenic SOD1^G93A^ mice and ALS patients, and may contribute to regulate inflammatory features of microglia and serve as a disease biomarker [51, 52]. Thus, the proteomic data obtained from mutant SOD1^G93A^ spinal cord is coherent with reported changes in ALS tissue. Moreover, the actin cytoskeleton regulators Filamin C (Flnc) and Vimentin (Vim) were also induced in SOD1^G93A^ mice (Additional file 1: Fig. S6). Mutations in Flnc have been linked to myopathy [53, 54], while Flnc up-regulation has been reported in brain tissue of frontotemporal lobar degeneration with TDP-43 inclusions (FTLD-TDP) patients [55]. The overexpression of ERp57 had minor effects on proteins modulated by mutant SOD1^G93A^, with 14 out of 23 hits also having significant changes in double transgenic mice (Fig. 4h and Additional file 1: Fig. S6; see Additional file 2: Table S2 for the complete set of quantified proteins).

Interestingly, Fasciculation and elongation protein zeta-1 (Fez1) and Disheveled-associated activator of morphogenesis 1 (Daam1), two down-regulated proteins in mutant SOD1^G93A^ involved in neuronal morphology and actin cytoskeleton organization, respectively, were rescued in double transgenic mice (Additional file 1: Fig. S6). The analysis of ERp57^WT^ transgenic mice revealed evident proteomic hits that were also observed in double transgenic animals when compared to non-Tg littermates (Fig. 4g, h and Additional file 1: Fig. S6). From these proteomic modifications, we highlight Ras-related GTP-binding protein B (Rragb, which responds to starvation), and regulating synaptic membrane exocytosis protein 3 (Rims3), a synaptic component previously found transcriptionally induced in the brain cortex of sALS patients [50]. Furthermore, ERp57 overexpression in ALS mice led to the induction of Erbin, a regulator of synaptic transmission at the NMJ, and Myristoylated alanine-rich C-kinase substrate (Marcks), another protein involved in actin cytoskeleton control (Additional file 1: Fig. S6). We also observed enhanced expression of Marcks in spinal cord from double transgenic mice using western blot, confirming the proteomic analysis (Additional file 1: Fig. S7). These results suggest the occurrence of quantitative changes at the proteomic level triggered by ERp57 that might contribute to improve synaptic function in mutant SOD1 mice. Further studies are needed to address the significance of these proteins to the neuroprotective effects of ERp57.

## Discussion

PDIs are major oxidoreductases that catalyze disulfide bond formation, reduction and isomerization in the ER, promoting the folding and quality control of membrane and secreted proteins [56]. The impairment of PDIs function may have important implications for ALS pathogenesis and other neurodegenerative diseases [57, 58]. Inactivation of PDI by S-nitrosylation has been found in spinal cord of ALS mouse models and *post-mortem* tissue of sALS and fALS patients [57, 59]. PDI S-nitrosylation has been correlated with the aggregation of mutant SOD1 in vitro and in the spinal cord of ALS mouse models [57, 60]. Silencing of PDI or ERp57 expression enhances mutant SOD1 aggregation in neuronal cell culture [19, 59], whereas PDI and ERp57 overexpression decreases mutant SOD1 aggregates in vitro [11, 19, 59]. Moreover, PDI co-localizes with inclusions of ALS-linked mutant proteins like FUS, VAPB and TDP43 [61–63]. A recent report also suggested that PDI overexpression can improve motor performance in a zebrafish model of ALS, although the mechanism of action was not investigated [11]. Based on this evidence, PDIs up-regulation is proposed as a neuroprotective response in ALS that may alleviate the burden of misfolded and aggregated proteins and reduce ER stress [18, 19]. All this evidence prompted us to study the consequences of overexpressing ERp57 in the progression of experimental ALS.

We have approached this problem by crossing a transgenic line overexpressing human ERp57 with the mutant SOD1^G93A^ mouse model. Contrary to the expectations based on the current literature, overexpression of ERp57 did not reduce motoneurons loss or extend lifespan of ALS mice. Rather, double transgenic SOD1^G93A^/ERp57^WT^ mice showed delayed deterioration of motor performance when clinical symptoms were already apparent. Interestingly, our results temporally dissected the effects of ERp57 on NMJ function from its possible role in mutant SOD1 aggregation [64–66]. Although we corroborated that ERp57 overexpression reduces mutant SOD1 aggregates in NSC-34 cells and late-stage mutant SOD1 mice, this phenomenon was not replicated at early-symptomatic stages. Thus, the protection afforded by ERp57 overexpression over the NMJ is likely to be unrelated to the modulation of abnormal protein aggregation, suggesting that distinct molecular mechanisms operate in ALS pathophysiology depending on the disease stage.

Regarding motoneuron physiology, double transgenic mice exhibited improved electrical activity and morphological integrity of NMJ, showing significantly higher CMAP values and reduced muscle denervation compared to SOD1^G93A^ littermates. These results are in accordance to our previous study suggesting a role for ERp57 on NMJ maturation under non-disease conditions [17]. However, it was unknown whether ERp57 overexpression could protect from the deterioration observed in ALS. While SOD1^G93A^ transgenic mice develop a pronounced CMAP decay already at 8 weeks of age, the extent of NMJ impairment at early-symptomatic disease stage was insufficient to cause motor dysfunction in our and other studies [67, 68]. The motor impairment detected at later time points may be due to a second wave of CMAP decay, possibly due to further pruning of innervation of different pools of motoneurons [67]. Despite the early protection of NMJ, the effects of ERp57 in motoneurons appear to be transient and not sufficient to slow disease progression at the global level or enhance survival. This observation may be related to additional pathological mechanisms that alter NMJ biology beyond dysfunction of ER proteostasis in motoneurons, as well as redox inactivation of ERp57, as reported for PDI [57, 59, 60]. Indeed, we observed that ERp57 reduces end-stage mutant SOD1 aggregation, possibly through intermediacy of mixed disulfide crosslinks that compromise its enzymatic activity. Furthermore, in our transgenic model ERp57 is predicted to have negligible interference on cell non-autonomous mechanisms that drive neurodegenerative cascades [3]. In fact, we have shown that overexpression of ERp57 enhances axonal regeneration and locomotor recovery after sciatic nerve damage in mice, but not dopaminergic neuron loss in a model of Parkinson’s disease, illustrating contrasting outcomes of the same genetic manipulation in different neurodegenerative contexts [25]. In addition, ERp57 overexpression in the brain was unable to modify the up-regulation of UPR markers in a pharmacological paradigm of ER stress [24]. In the case of experimental ALS, the damaging tissue environment could explain the unaltered disease progression in double transgenic mice, despite the protection of motoneuron physiology exerted by ERp57 overexpression. This neuroprotective action of ERp57 at the nerve terminal of mutant SOD1^G93A^ mice is predicted to result from improved ER proteostasis enhancing the folding and expression of proteins composing NMJ, since ERp57 deficiency in the nervous system leads to altered folding of certain synaptic proteins [17]. Accordingly, we reported that ERp57 expression augments steady-state levels of the Prion protein [24], a factor involved in axonal growth and synaptic function [69, 70].

To determine possible molecular mechanisms of NMJ protection, we performed an unbiased approach with proteomic analysis of lumbar spinal cord at early-symptomatic stage of the disease. Induction of Mycbp2 in SOD1^G93A^ model along with the actin cytoskeleton regulators FlnC and Vim corresponded to major proteomic alterations detected likely reflecting a motoneuron response to cope with axonal damage and/or NMJ denervation. Importantly, Rims3 has been shown to be up-regulated in tissue of sALS patients along with other synaptic proteins [50]. The role of these proteomic hits in ALS requires further investigation, in addition to defining the mechanisms that explain their modulation when ERp57 is overexpressed. Overall, our results suggest that the upregulation of ERp57 in observed ALS may represent an adaptive response to sustain NMJ in this pathological context (see model in Fig. 4i).

## Conclusions

Dysregulation of ER proteostasis is emerging as a transversal pathogenic mechanism in ALS. Previous studies have suggested that strategies to up-regulate the ER oxidoreductase ERp57 in ALS may have therapeutic value by improving the adaptive reaction against protein aggregation and ER stress. Here, we defined the significance of ERp57 to ALS pathophysiology in vivo and demonstrated that ERp57 exerts neuroprotective roles associated to improved NMJ stability and function, a phenomenon that may be dissociated from SOD1 aggregation. Since strategies to strengthen NMJ may prove key to maintain motor capacity in ALS, the combination of ERp57 overexpression using gene therapy or pharmacological approaches with interventions to tackle other pathogenic mechanisms may pave the way for future translational development.

## Supplementary Information

**Additional file 1**: Supplementary Data and Related Materials and Methods.

**Additional file 2**: Proteomics Dataset.

## Data Availability

Raw datasets and analysis of proteomic experiment can be found in supplementary information Table S2. The mass spectrometry proteomics raw data is available at the ProteomeXchange Consortium. All datasets are available from the corresponding author upon reasonable request.

## Abbreviations

ALS: amyotrophic lateral sclerosis
sALS: sporadic ALS
fALS: familial ALS
ANOVA: analysis of variance
CMAP: compound muscle action potential
DTT: dithiothreitol
ER: endoplasmic reticulum
HMW: high molecular weight species
MND: motor neurone disease
NMJ: neuromuscular junction
PCR: polymerase chain reaction
PDIs: protein disulfide isomerases
PVDF: polyvinylidene difluoride
RT: room temperature
RT-qPCR: quantitative reverse transcription PCR
SDS: sodium dodecyl sulfate
SDS-PAGE: polyacrilamide gel electrophoresis with SDS
SNP: single nucleotide polymorphism
UPR: unfolded protein response
