# Supplementary material for: Protein disulfide isomerase ERp57 protects early muscle denervation in experimental ALS
Source: Acta Neuropathol Commun. 2021 Feb 4;9:21. doi: 10.1186/s40478-020-01116-z (PMC7863244; doi:10.1186/s40478-020-01116-z)
Supplement: Supplementary file 1 — Additional file 1: Supplementary Data and Related Materials and Methods. [file 40478_2020_1116_MOESM1_ESM.pdf]

Supplementary Information for

Protein disulfide isomerase ERp57 protects early muscle denervation in experimental ALS

Pablo Rozas[1,2,3], Cristina Pinto[4], Francisca Martínez Traub[1,2,3], Rodrigo Díaz[1,2,3], Viviana Pérez[4], Daniela Becerra[1,2,3], Patricia Ojeda[1,2,3], Jorge Ojeda[4], Madison T. Wright[5], Jessica Mella[4], Lars Plate[5], Juan Pablo Henríquez[4], Claudio Hetz[1,2,3,6*] and Danilo B. Medinas[1,2,3*]

[1]*Biomedical Neuroscience Institute, Faculty of Medicine, University of Chile, Santiago, Chile*

[2]*Center for Geroscience, Brain Health and Metabolism, Santiago, Chile*

[3]*Program of Cellular and Molecular Biology, Institute of Biomedical Sciences, University of Chile, Santiago, Chile*

[4]*Neuromuscular Studies Laboratory (NeSt Lab), Department of Cell Biology, Faculty of Biological Sciences, Center for Advanced Microscopy (CMA Bio-Bio), Universidad de Concepción, Concepción, Chile*

[5]*Department of Chemistry and Department of Biological Sciences, Vanderbilt University, Nashville, TN, USA*

[6]*Buck Institute for Research on Aging, Novato, CA, USA*

[*]Danilo B. Medinas: dmedinas@med.uchile.cl and Claudio Hetz: chetz@uchile.cl, chetz@buckinstitute.org.

This PDF file includes:

Supplementary Materials and Methods
Figure S1 to S7
Table S1

Supplementary Information Text

Materials and Methods

Phenotypic characterization. Disease progression of $SOD1^{G93A}$ ALS model was followed using body weight measurements, wire hang test, rotarod test, and clinical score analysis. Body weight was measured once a week starting at 5 weeks of age until end point.

Rotarod test was conducted by placing the mouse in a rotating cylinder under constant acceleration protocol from 4 to 40 rpm in 2 min. Latency to fall from the cylinder to the base platform was recorded as measure of motor performance. This protocol assures the fall of mice due to limb problems and not from stamina draining. One week before rotarod test, mice were trained for 5 days by walking on the cylinder at a constant speed of 4 rpm for 1 minute followed by 10 rpm for another minute. Rotarod test was performed in a single session of 3 trials once a week starting at six weeks of age. Mice that failed learning the test during the training period were excluded from analysis.

Clinical score was determined by assessing four disease parameters quantified according to an arbitrary scale: 1) Hindlimb clasping: under healthy conditions mice fully extend their hindlimbs when hold by the tail, while symptomatic mice are unable to extend the hindlimbs; 2) Kyphosis: curvature of the spine; 3) Absence of grooming: observed as dirty fur and eyes; 4) Paralysis: starting as difficulty to move hindlimbs during cage walking. For each parameter, the score was assigned depending on the severity: 0: absence of sign; 1: mild sign; 3: moderate sign; 5: severe sign. Clinical score was measured once a week starting at 5 weeks of age and its value corresponded to the sum of each parameter. Measurements of clinical score were performed by an observer blinded to the genotype to avoid bias.

Lumbar spinal cord histological analysis. For total motoneuron count, 16 serial sections from lumbar spinal cord per animal were used assuring 3.2 mm coverage of the lumbar segment of the spinal cord from L5 to L2 (one section every 200 μm). For gliosis analysis, 4 serial sections from lumbar spinal cord per animal were used assuring 3.2 mm coverage of the lumbar segment of the spinal cord from L5 to L2 (one section every 800 μm).

For total motoneuron count, anti-ChAT immunohistochemistry (IHQ) was performed by treating spinal cord sections with 3% H_2O_2 - 10% MeOH in TBS for 15 min at RT to inhibit endogenous peroxidases. After one wash step with TBS, epitope retrieval was performed using citrate buffer (10 mM sodium citrate, 0.05% Tween 20, pH 6.0) for 5 min at 95°C. After one wash with TBS, spinal cord cryosections were mounted on super frost slides (VWR International). Sections were blocked using 5% donkey serum diluted in 0.2% Triton X-100 in TBS (blocking buffer) for 1 h at room temperature (RT) and then incubated with 1:250 anti-ChAT antibody (MerckMillipore, AB144P) in blocking buffer for 48 h at RT. Sections were then washed with TBS six times for 15 min each and incubated with 1:2,000 anti-goat HRP-conjugated antibody (MerckMillipore, AP180P) in blocking buffer for 3 h at RT and then washed with TBS six times for 15 min each. Chromogenic reaction was performed using DAB kit (Vector Lab) following manufacturer's instructions. ChAT positive cells were manually counted using bright field microscopy.

For astrocyte and microglia activation, IF analysis using anti-GFAP antibody (Abcam, ab7260) or anti-Iba1 antibody (Wako, 019-19741) was performed, respectively. Epitope retrieval was performed using citrate buffer (10mM sodium citrate, 0.05% Tween 20, pH 6.0) for 5 min at 95°C. After one wash with Phosphate-buffered saline (PBS), spinal cord cryosections were mounted on super frost slides (VWR International).

Sections were blocked using 1% BSA diluted in 0.025% Triton X-100 in PBS for 2 h at RT and then incubated with 1:500 anti-GFAP or 1:500 anti-Iba1 antibodies in 1% BSA in PBS overnight at 4°C. Sections were then washed four times for 5 min each and incubated with 1:1,000 anti-rabbit Alexa-488 conjugated secondary antibody (Molecular Probes), and 1:5,000 Hoechst 33,342 for nuclear staining, in 1% BSA in PBS for 2 h at RT. After four washes in PBS, sections were covered with coverslips using Fluoromount-G (Thermo Fisher Scientific) as mounting medium. Confocal microscopy (Nikon eclipse C2+) was used to obtain micrographs of both ventral horns per section. Alexa-488 staining area in ventral horn was quantified using ImageJ (NIH, Bethesda, Maryland). Automatic background subtraction was performed using ImageJ custom macro for all GFAP or Iba1 set of images. This macro was set up using symptomatic $SOD1^{G93A}$ and non-Tg technical control L5 sections.

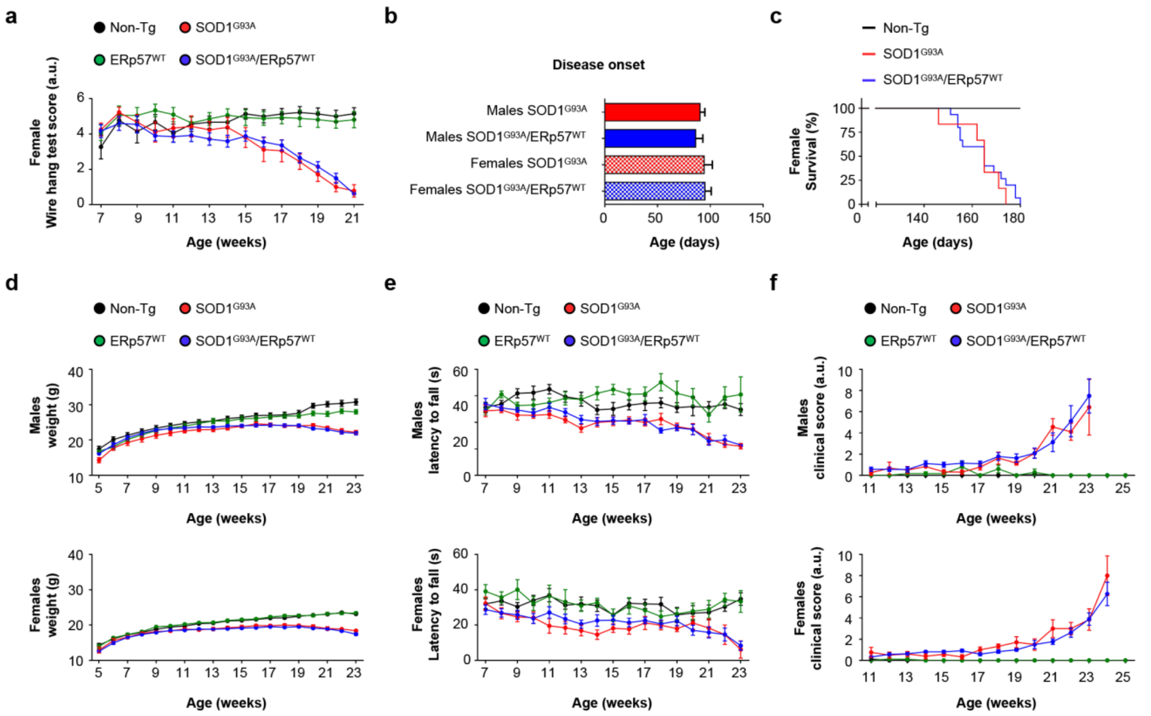

Fig. S1. Phenotypic characterization of $SOD1^{G93A}/ERp57^{WT}$ mice by gender. a Wire hang test performance. Statistical analysis was performed using two-way ANOVA with Tukey's multiple comparison test. Mean ± S.E. is shown (n = 7-16 female mice per genotype). **b** Analysis of disease onset according to motor performance in the wire hang test. Disease onset was defined as the time point when mice started to lose performance and determined for each animal individually. Statistical analysis was performed using one-way ANOVA with Tukey's multiple comparison test. Mean ± S.E. is shown (n = 7-16 mice per genotype). **c** Survival of double transgenic mice compared to $SOD1^{G93A}$ group. Non-transgenic (Non-Tg) animals were plotted as control (n = 6-15 female mice per genotype). **d** Body weight measurements. Statistical analysis was performed using two-way ANOVA with Tukey's multiple comparison test. Mean ± S.E. is shown (n = 6-14 male mice per genotype; n = 7-16 female mice per genotype). **e** Rotarod test performance. Statistical analysis was performed using two-way ANOVA with Tukey's multiple comparison test. Mean ± S.E. is shown (n = 6-12 male mice per genotype; n =

7-14 female mice per genotype). **f** Clinical score analysis. Statistical analysis was performed using two-way ANOVA with Tukey's multiple comparison test. Mean ± S.E. is shown (n = 6-14 male mice per genotype; n = 7-16 female mice per genotype).

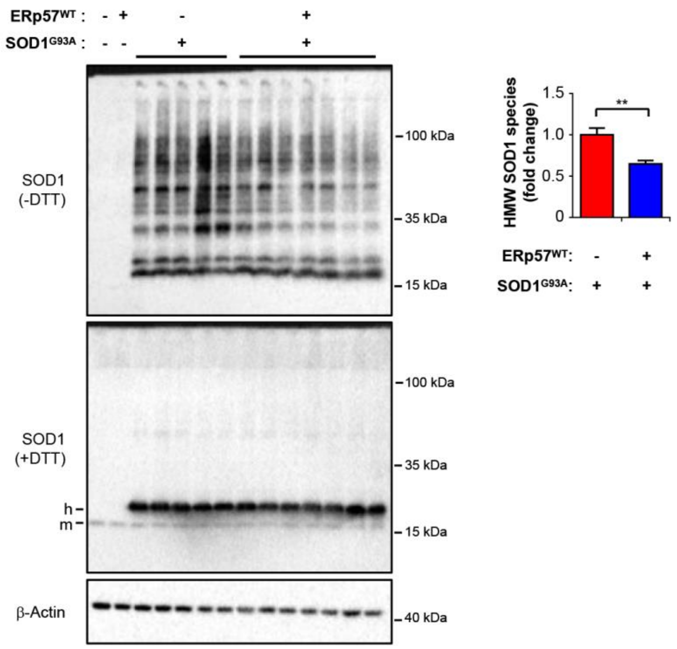

Fig. S2. Analysis of SOD1 high molecular weight species. Western blot analysis from end-stage lumbar spinal cord extracts. Left panel: SOD1 high molecular weight (HMW) species were assessed under non-reducing condition (without the reducing reagent DTT, -DTT). Total SOD1 levels were assessed under reducing condition (+DTT). Anti-SOD1 antibody binds to human (h) and mouse (m) SOD1. One non-transgenic and one $ERp57^{WT}$ single transgenic age-matched mice were included as control. β-actin was employed as loading control. Each lane corresponds to one animal. SOD1 HMW species were quantified by densitometric analysis of bands at 35 kDa and above, including the signal corresponding to the stacking gel. Statistical analysis was performed using Student's *t*-test comparing $SOD1^{G93A}$ and double transgenic animals. Mean ± S.E. is shown. *p* value: **, $p \leq 0.01$.

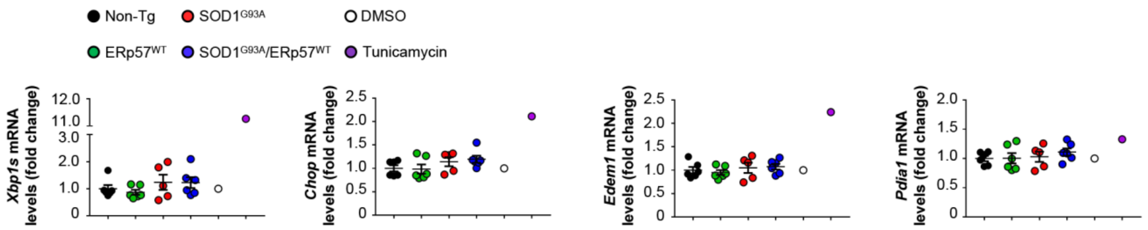

Fig. S3. Evaluation of the unfolded protein response in lumbar spinal cord at early-symptomatic stage. Gene expression analysis of genes related to the unfolded protein response (UPR) was performed by RT-qPCR at post-natal day 90. Each gene is normalized to non-transgenic control group (Non-Tg). Statistical analysis was performed using one-way ANOVA with Tukey's multiple comparison test. Mean ± S.E. is shown; no differences were observed (n = 5-6 mice per genotype). Each dot corresponds to one animal. Spinal cord samples from mice treated for 24 h with tunicamycin (5 mg/kg) or vehicle (dimethyl sulfoxide, DMSO) by intraperitoneal injection were used as technical controls of UPR activation.

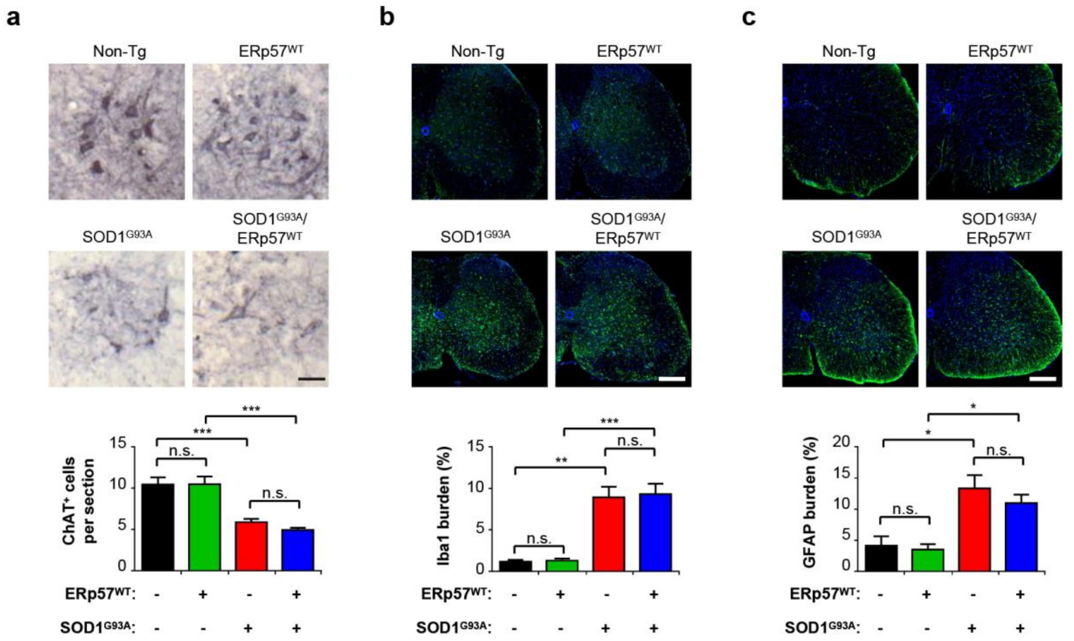

Fig. S4. Histopathological characterization of SOD1^{G93A}/ERp57^{WT} lumbar spinal cord at end-stage. a Analysis of motoneuron number by anti-ChAT immunohistochemistry. Graph shows average motoneuron number quantified from 16 serial sections spanning L2 to L5. **b** Analysis of microglia activation by anti-Iba1 immunofluorescence (pseudocolored green). Graph shows average of Iba1 positive area in ventral horn grey matter quantified from 4 serial sections spanning L2 to L5 and expressed as Iba1 burden. **c** Analysis of astrocytes activation by anti-GFAP immunofluorescence (pseudocolored green). Graph shows average of GFAP positive area in ventral horn grey matter quantified from 4 serial sections spanning L2 to L5 and expressed as GFAP burden. Cell nuclei were staining with Hoechst 33,342 (pseudocolored blue) in **b** and **c**. Statistical analysis was performed using one-way ANOVA with Tukey's multiple comparison test in **a**, **b** and **c**. Mean ± S.E. is shown; *p* values: n.s., $p > 0.05$; *, $p \leq 0.05$; **, $p \leq 0.01$ and ***, $p \leq 0.001$ (n = 3-8 animals per genotype). Scale bars: **a** 50 μm; **b** 250 μm; **c** 250 μm.

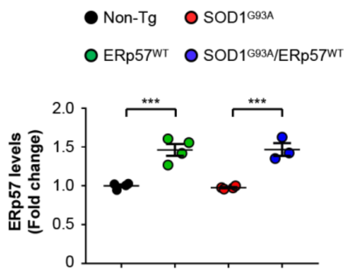

Fig. S5. Analysis of ERp57 protein expression. Protein extracts from lumbar spinal cord of mice at post-natal day 90 were analyzed by mass spectrometry proteomic approach. Fifteen unique peptides were identified for ERp57, seven conserved between human and mouse species and eight corresponding to the mouse protein sequence only. Relative protein abundance of ERp57 was calculated by the sum of intensities of conserved peptides normalized by mouse only peptides. The expression is shown as fold change relative to non-Tg group. Statistical analysis was performed using one-way ANOVA with Tukey's multiple comparison test. Mean ± S.E. is shown; *p* value: ***, $p \leq 0.001$ (n = 3-4 mice per genotype). Each dot corresponds to one animal.

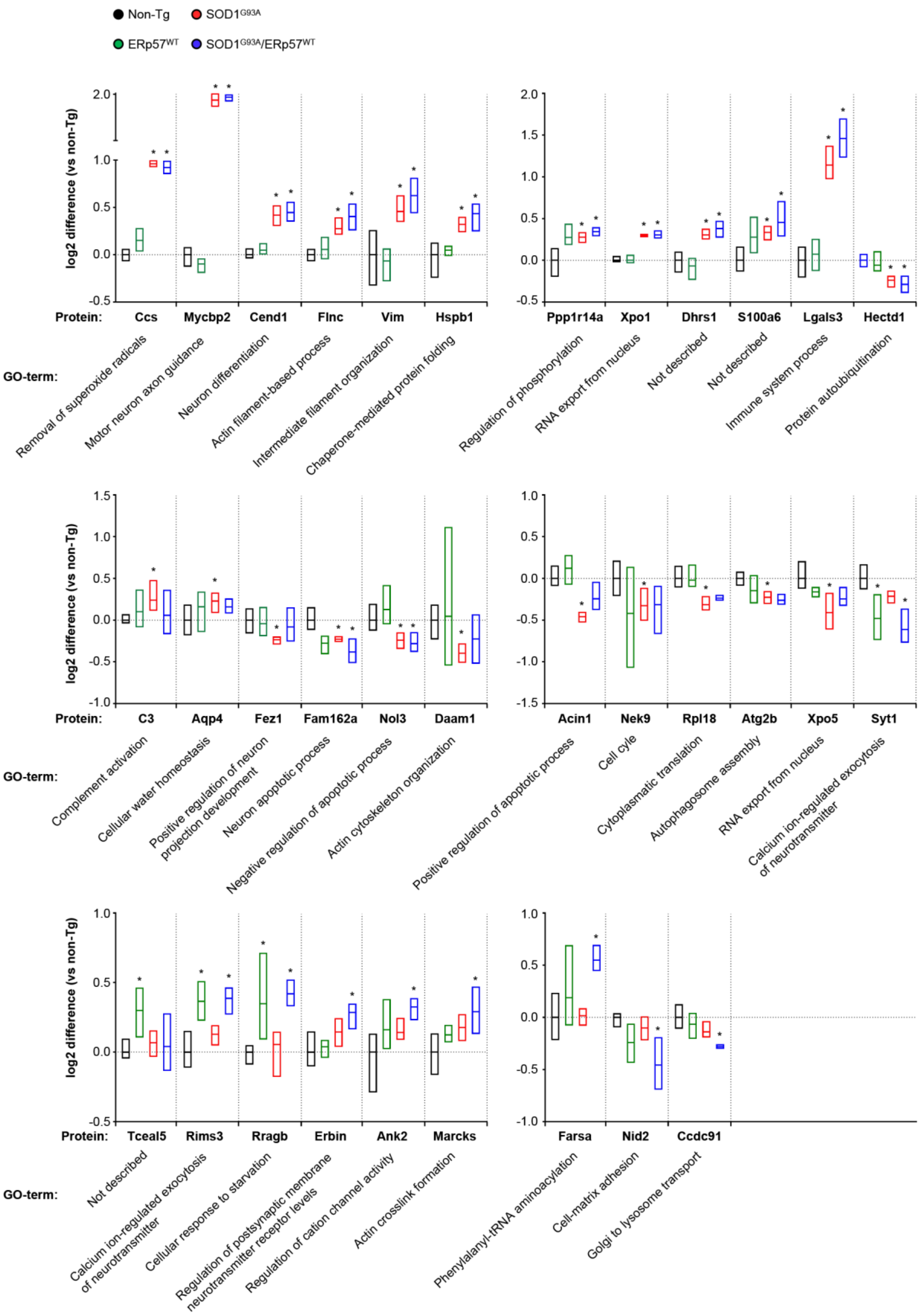

Fig. S6. Box plots of proteomic hits from early-symptomatic lumbar spinal cord. Proteomic analysis was performed from lumbar spinal cord of mice at post-natal day 90.

Each box shows minimum, maximum and median values for each genotype. Differences in protein levels are shown relative to non-transgenic littermates (non-Tg). Gene Ontology term (GO-term) corresponds to biological process category assigned to each hit. Statistical analysis was performed using multiple t-test with two-stage step-up method using Benjamini, Krieger and Yekutieli approach with a False Discovery Rate of 5%. Asterisk (*) indicates hits with q-value ≤ 0.05 compared to non-Tg group (n = 3-4 animals per genotype).

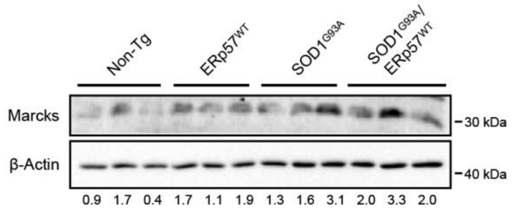

Fig. S7. Validation of Marcks protein levels at early-symptomatic stage. Western blot analysis was performed from lumbar spinal cord of mice at post-natal day 90. Each lane corresponds to a different animal. The β-actin blot shown as loading control is the same of Fig. 4d. Quantification of Marcks band intensity for each sample is shown as fraction relative to non-Tg mean.

Table S1. Animals generated from crossing ERp57^{WT} and SOD1^{G93A} transgenic lines.

	**Non-Tg**		**ERp57^{WT}**		**SOD1^{G93A}**		**SOD1^{G93A}/ERp57^{WT}**		**Total**
**Observed**	**Males**	**Females**	**Males**	**Females**	**Males**	**Females**	**Males**	**Females**	
Count	17	17	9	11	15	10	11	18	108
%	15.7	15.7	8.3	10.2	13.9	9.3	10.2	16.7	100
Total %	**31.5**		**18.5**		**23.1**		**26.9**		**100**
**Expected**									
%	12.5	12.5	12.5	12.5	12.5	12.5	12.5	12.5	100
Total %	**25**		**25**		**25**		**25**		**100**
